# The effectiveness of ginger extract addition in calorified drinks during perioperative period to nausea severity, vomitus, post-operative anxiety, and metabolic disorder: A randomized control trial

**DOI:** 10.1016/j.amsu.2022.104865

**Published:** 2022-11-11

**Authors:** Aaron Tigor Sihombing, Diaswara Prabharani, Kiki Lukman, Reza Widianto Sudjud

**Affiliations:** a Medical Education, Development, and Innovation Center University Padjadjaran, Indonesia; b Department of Urology, Hasan Sadikin Hospital, Bandung, Universitas Padjadjaran Bandung, Indonesia; c Department of Digestive Surgery, Hasan Sadikin Hospital, Bandung, Universitas Padjadjaran Bandung, Indonesia; d Department of Anesthesiology and Intensive Care, Hasan Sadikin Hospital, Bandung, Universitas Padjadjaran Bandung, Indonesia

**Keywords:** Anxiety, Ginger extract, High calorie drink, Nausea, Preoperative vomiting

## Abstract

**Background:**

This study was aimed to evaluate the effect of ginger extract addition to pre-operative high calorie drink to reduce nausea, vomiting, anxiety level, and lactate level as a parameter for metabolic derangement in patients undergoing surgery.

**Materials and methods:**

A prospective single-blinded randomized controlled trial design, with a study subject elective surgical procedures patient at the Department of Surgery, Dr Hasan Sadikin Hospital, Bandung. Patient were divided into two groups, group A with standard high calorie drink, and group B standard high calorie drink with ginger extract addition. Anxiety was measured by HARS score and the occurrence of nausea and vomiting were assessed after the operation. The difference of lactate levels was compared before and after operation in two groups. The data distribution was calculated by kolmogorov-smirnov for continues variables. Normal distribution was calculated by *t*-test, non-normal distribution was calculated by Mann-U Whitney. Nominal data calculated by chi-square test and ordinal data calculated by Mann-U Whitney.

**Results:**

40 subjects were randomly divided into two groups. The occurrence of nausea and vomiting were lower in group B patients (p < 0.0285). HARS score anxiety level was lowered in group B patients (p < 0.0293). Lactate level was lowered in group B patients, although is not statistically significant (p 0.54).

**Conclusion:**

Addition of ginger extract in pre-operative high calorie drink reduce postoperative nausea, vomiting, and anxiety level.

## Highlights

• The occurrence of nausea and vomiting were lower after high calorie ginger drink administration.
• HARS score anxiety level was lowered after high calorie ginger drink administration.
• Lactate level was lowered in average after high calorie ginger drink administration, but not statistically significant.
• Addition of ginger extract in pre-operative high calorie drink reduce postoperative nausea, vomiting, and anxiety level.

## 1 Introduction

During starvation, various metabolic changes occur in the body. One of the metabolic disorders might happened in the body was the disruption of the glucose consumption as the main energy source in cells [1]. Under conditions of normal perfusion and oxygen levels in the body, glucose was catabolized as and utilized as intracellular energy to form pyruvate. However, if there was impaired tissue perfusion which might causes an imbalance oxygen levels in cells, such as in a state of dehydration and hypoxia, anaerobic glycolysis will occur [2]. This situation is similar to hypoxia, which is a condition in which anaerobic glycolysis is activated thereby limiting the entry of pyruvate into the Tricarboxylic Acid (TCA) cycle. Alternatively, pyruvate is converted to lactate, reducing a 17-fold reduction in net ATP production [3].

Changes in metabolism during starvation, which results in the increasing of lactate levels have an impact on a personal mental condition. A study had discovered the effects of psychosocial and physical stress on lactate levels and anxiety levels, it was found that an increase in lactate levels was accompanied by the increasing of anxiety levels according to the severity of the stressor [4]. Anxiety could also trigger nausea. This could be seen from a phenomenon called Anticipatory Nausea and Vomiting (ANV). This ANV phenomenon is a condition where nausea and vomiting occur when anxiety occurs before chemotherapy is given. But on the other hand, nausea from some studies can also cause anxiety that triggers nausea and vomiting [5].

Enhanced Recovery After Surgery (ERAS), is a method currently used to reduce the incidence of nausea, vomiting, depression, postoperative anxiety and other factors that prolong the length of hospital stay [6]. Enhanced Recovery After Surgery (ERAS) is a dynamic element of perioperative care [6]. The strongest evidence of successful application of ERAS has been seen in nursing care of patients undergoing open colonic resection [7]. Many of the interventions that previously proved useful in this population have also been successfully applied to other surgical interventions, such as laparoscopic colon resection, as well as interventions in other surgical specialties such as urology, orthopedics, and gynecology [6].

Glucose intake before surgery, as one of the ERAS protocol, is expected to replace the loss of energy sources in patients who will undergo surgery. In a randomized study, administration of glucose solution reduced hunger, thirst and anxiety [8]. However, this preoperative glucose administration may be complicated by nausea and vomiting associated with the surgical patient's illness.

Ginger has traditionally been used to treat gastrointestinal symptoms, and recent research has shown that ginger can effectively relieve nausea and vomiting [9]. In a clinical trial, consuming ginger juice reduced the intensity of nausea and decreased episodes of emesis during the period of two to 6 h after nephrectomy in patients. In addition, treatment with dry ginger powder can reduce episodes of intraoperative nausea in patients with elective cesarean section [9]. Results from three RCTs with a total of 272 participants showed a statistically significant reduction in the incidence of vomiting with the use of *Zingiber officinale* compared with the control group (i.e. placebo and water) in laparoscopic surgery and obstetrics/gynecology patients [10].

An experimental in vitro study revealed that 6-shogaol, 6-gingerol, and zingerone inhibited the transmission of emetic signals in vagal afferent neurons by suppressing 5-HT, and 6-shogaol receptors had the strongest inhibitory effectiveness [9]. This study was aimed to assessed the effect of adding calories with ginger extract to reduce the incidence of nausea, vomiting, and reduce levels of anxiety, as well as its effect on metabolic disorders by looking at lactate levels in patients undergoing surgery.

## 2 Methods

This study used a prospective single-blinded randomized controlled trial design with a study subject of patients undergoing elective surgical procedures at the Department of Surgery, Dr Hasan Sadikin Hospital, Bandung who met inclusion and exclusion criteria. The inclusion criteria of the subject was patient who underwent elective surgery at the Department of Surgery, Dr Hasan Sadikin Hospital, Bandung; patient aged ≥18 years; and patient comply with ASA Grade 1 or 2. The exclusion criteria were including pregnancy, patient with swallowing disorder, patient with allergic history of certain foods (especially milk and ginger), and patient with vestibular disorder which might causes nausea and or motion sickness.

The variable being obtained during the study were age, gender, nausea, vomitus, severity of anxiety assessed by *Hamilton Anxiety Rating Scale* (HARS) score, and level of serum lactic acid. Normality test was being conducted for numerical data with *Kolmogorov Smirnov* test, the significance test to compare the numerical characteristics of the data from the two treatment groups was being conducted by using paired T test if the data was normally distributed and the Wilcoxon test as an alternative if the data was not normally distributed. The significance test to compare the descriptive categorical characteristics of the data from the two treatment groups used the Chi-square test. This study has been reported in line with the CONSORT criteria [11].

## 3 Results

There were 40 subjects who were divided into two groups, intervention group of patients with ginger extract drinks and control group of patients with placebo drinks. In the intervention group, 15 (75.0%) subjects were male and 5 (25.0%) subjects were female, the average age of 49.45 ± 16.41 years, and an even age distribution of each age group. In the control group, 16 (80.0%) subjects were male and 4 (20.0%) subjects were female with an average age of 45.90 ± 20.22 years and the highest age distribution being at the age group of 25–44 years as many as 8 (40.0%) subjects.

There were factors that were significantly affected by the administration of drinks with ginger extract which are the incidence of nausea with a value of p = 0.0285 (p < 0.05) and the degree of post-op anxiety based on the HARS score with a value of p = 0.0293 (p < 0.05). (Table 1).

**Table 1:** Characteristics and comparison of the incidence of nausea, vomiting, anxiety, and serum lactic acid levels in the two treatment groups of patients.

Variable	Treatment				p-value
	Ginger		Placebo		
	n	%	n	%	
Sex					
Male	15	75,0	16	80,0	
Female	5	25,0	4	20,0	
Age					
18–24 years	5	25,0	1	5,0	
25–44 years	5	25,0	8	40,0	
45–60 years	5	25,0	6	30.0	
≥60 years	5	25,0	5	25,0	
Age (mean ± std)	49,45 ± 16,41		45,90 ± 20,22		
Nausea					**0,0285**
Yes	2	10,0	8	40,0	
No	18	90,0	12	60,0	
Vomitus					0,3758
Yes	2	10,0	4	25,0	
No	18	90,0	16	75,0	
Pre-op anxiety severity pre-op based on HARS scoring system					0,1572
No anxiety (<14)	18	90,0	14	70,0	
Mild anxiety (14–20)	2	10,0	3	15,0	
Moderate anxiety (21–27)	0	0	3	15,0	
Severe anxiety (28–41)	0	0	0	0	
Very severe anxiety (42–56)	0	0	0	0	
Post-op anxiety severity pre-op based on HARS scoring system					**0,0293**
No anxiety (<14)	20	100,0	14	70,0	
Mild anxiety (14–20)	0	0	3	15,0	
Moderate anxiety (21–27)	0	0	3	15,0	
Severe anxiety (28–41)	0	0	0	0	
Very severe anxiety (42–56)	0	0	0	0	
Lactic acid level (mean ± std)	2,52 ± 1,42		2,82 ± 1,53		0.5414

In the group of patients with intervention of ginger extract addition, 2 (10.0%) people experienced nausea and vomiting, while 18 (90%) people did not experience nausea and vomiting. Meanwhile, in the group of patients treated with placebo drinks, 8 (40.0%) people experienced nausea and 12 (60.0%) people did not experience nausea, while 4 (25.0%) people experienced vomiting and 16 (75.0%) experienced nausea. (0%) people do not experience vomiting.

Anxiety degrees were assessed based on the HARS scoring system before (pre-op) and after (post-op) surgery. In the group of patients treated with drinks with the addition of ginger extract, 18 (90.0%) patients did not experience pre-op anxiety and 2 (10.0%) patients experienced mild pre-op anxiety. Meanwhile, in the group of patients treated with placebo drinks, 14 (70.0%) patients did not experience pre-op anxiety, 3 (15.0%) patients experienced mild pre-op anxiety, and 3 (15.0%) had moderate pre-op anxiety.

In the group of patients treated by drinks with the addition of ginger extract, 20 (100.0%) people did not experience post-op anxiety and 14 (70.0%) people did not experience post-op anxiety, 3 (15.0%) people experienced mild post-op anxiety, and 3 (15.0%) experienced moderate post-op anxiety. In the group of patients treated by drinks with the addition of ginger extract, the serum lactic acid level was 2.52 + 1.42 and the serum lactic acid level in the placebo patient was 2.82 + 1.53.

## 4 Discussion

Obtained factors that were significantly affected by the administration of drinks with ginger extract which are the incidence of nausea with a value of p = 0.0285 (p < 0.05) and the degree of post-op anxiety based on the HARS score with a value of p = 0.0293 (p < 0.05). These results are in accordance with a study conducted by Zeraati et al. [11] which determined that according to an independent *t*-test, there was a significant relationship in the group of patients with the intervention of ginger extract drinks and the control group of patients in the incidence and mean severity score of nausea and vomiting during caesarean section. (P < 0.05) [12].

Ginger dramatically reduced nausea and vomiting sensations more than vitamin B6, which is a traditional first-line treatment for nausea, according to a new study comparing the two treatments [13]. The incidence of acute, but not delayed, nausea was considerably decreased by all concentrations of ginger, with 0.5 and 1.0 g being the most beneficial, according to the results of mixed-model analysis by Ryan et al. [14].

The main pharmacological activity of ginger was caused by the presence of gingerols and shogaols and the relative proportions of gingerols, shogaols, and paradols. Other constituents include capsaicin, gingediol, galanolactone, gingesulfonic acid, galactosylglycerols, gingerglycolipids, diarylheptanoids, neral, and phytosterols. Ginger gives advantages to digestive tract, by increasing bile secretion and preventing gastric ulcers [15,16].

Ginger has been shown to have strong antiemetic properties, but until now its mechanism of action has not been clearly elucidated. Among the active substances, [6]-, [8]-, and [10]-gingerol and [6]-shogaol have been shown in different in vivo studies to be partially responsible for their antiemetic properties. To gain more insight into the mechanism of action of the compound, 3 different in vitro models were used to investigate its effect on the 5-HT receptor (3) (serotonin receptor subtype). It was observed that [6]-, [8]-, [10]-gingerol, as well as [6]-shogaol exert their antiemetic effects at least in part by acting on the 5-HT(3) receptor ion channel complex, by binding to the modulating site associated with it. Differ from serotonin binding sites, including by indirect effects through receptors in signaling cascades behind the 5-HT receptor (3) channel complexes such as substance P receptors and muscarinic receptors. Therefore, its action can be summarized as 5HT3 antagonist activity, NKI antagonist, antihistamine and prokinetic effects without any side effects [17].

In this study, obtained in the group of patients treated with drinks with the addition of ginger extract, the serum lactic acid level was 2.52 ± 1.42 and the serum lactic acid level in the placebo patient was 2.82 ± 1.53. There was a relative increase in serum lactic acid levels in patients given placebo compared to patients given ginger extract, although this proved not to be significant. In the study of Toda et al., it was suggested that black ginger extract increased the uptake of 2-deoxyglucose and lactic acid as well as the mRNA expression of glucose transporter (GLUT) 4 and monocarboxylate transporter (MCT) 1 in both cell types. The expression of peroxisome proliferator-activated coactivator c receptor (PGC)-1a was increased in pC2C12 cells. In addition, black ginger extract increases ATP production and mitochondrial biogenesis. The polymethoxy flavonoids in black ginger extract include 5-hydroxy-7-methoxyflavone, 5-hydroxy-3,7,40-trimethoxyflavone and 5,7-dimethoxyflavone increased the expression of GLUT4 and PGC-1a. In addition, black ginger extract and 5,7-dimethoxyflavone increased the phosphorylation of 50AMP kinase-activated protein (AMPK) [18].

In this study, it was found that the addition of ginger extract had a significant effect on the degree of post-op anxiety based on the HARS scoring with a value of p = 0.0293 (p < 0.05). Changes in metabolism in a state of starvation, which results in increased lactate levels have an influence on a person's mental condition. From one of the studies that studied the effects of psychosocial and physical stress on lactate levels and anxiety levels, it was found that an increase in lactate levels was accompanied by an increase in anxiety levels according to the severity of the stressor [5].

## 5 Conclusion

The addition of ginger extract to calorified drinks in the perioperative period had a significant effect on reducing the incidence of nausea, but did not significantly reduce the incidence of perioperative vomiting. The addition of ginger extract to calorified drinks in the perioperative period had a significant effect on reducing postoperative anxiety. The addition of ginger extract to calorie drinks in the perioperative period was able to reduce post-operative lactic acid levels although it had no significant effect.

## Ethical approval

This study was approved by ethical committee of Faculty of Medicine Universitas Padjadjaran and Hasan Sadikin Hospital, Bandung Indonesia.

## Sources of funding

The authors received no financial support for the research, authorship, and/or publication of this article.

## Author contribution

Aaron Tigor Sihombing made contribution in the conception and design of the study. Diaswara Prabharani took part in acquisition of data, analysis and interpretation of data. Kiki Lukman drafted the article and revised it. Reza Widianto Sudjud approved the final version to be submitted.

## Trial registry number

1. Name of the registry:

2. Unique Identifying number or registration ID:

3. Hyperlink to your specific registration (must be publicly accessible and will be checked):

## Guarantor

Aaron Tigor Sihombing.

## Consent

Written informed consent was obtained from the patient for publication of this case report and accompanying images. A copy of the written consent is available for review by the Editor-in-Chief of this journal on request.

## Provenance and peer review

Not commissioned, externally peer-reviewed.

## Declaration of competing interest

The authors declare that they have no conflicts of interest related to this publication.
